# Laser-Synthesized SERS Substrates as Sensors toward Therapeutic Drug Monitoring

**DOI:** 10.3390/nano9050677

**Published:** 2019-05-01

**Authors:** Matteo Tommasini, Chiara Zanchi, Andrea Lucotti, Alessandro Bombelli, Nicolò S. Villa, Marina Casazza, Emilio Ciusani, Ugo de Grazia, Marco Santoro, Enza Fazio, Fortunato Neri, Sebastiano Trusso, Paolo M. Ossi

**Affiliations:** 1 Dipartimento di Chimica, Materiali e Ingegneria Chimica “G. Natta”, Politecnico di Milano, Piazza Leonardo da Vinci 32, 20133 Milano, Italy; matteo.tommasini@polimi.it (M.T.); chiaragiuseppina.zanchi@polimi.it (C.Z.); andrea.lucotti@polimi.it (A.L.); nicolosimone.villa@mail.polimi.it (N.S.V.); 2 Dipartimento di Energia, Politecnico di Milano, Via Ponzio 34/3, 20133 Milano, Italy; alessandro.bombelli@mail.polimi.it; 3 Division of Neurophysiopathology, Fondazione IRCCS Istituto Neurologico Carlo Besta, Via Celoria 11, 20133 Milano, Italy; marina.casazza@istituto-besta.it; 4 Laboratorio di Patologia Clinica e Genetica Medica, Fondazione IRCCS Istituto Neurologico Carlo Besta, Via Celoria 11, 20133 Milano, Italy; emilio.ciusani@istituto-besta.it (E.C.); ugo.degrazia@istituto-besta.it (U.d.G.); 5 Dipartimento di Scienze Matematiche e Informatiche, Scienze Fisiche e Scienze della Terra, Università di Messina, Viale Ferdinando Stagno d’Alcontres 31, 98166 Messina, Italy; masantoro@unime.it (M.S.); enfazio@unime.it (E.F.); fneri@unime.it (F.N.); 6 CNR-IPCF, Istituto per i Processi Chimico-Fisici del CNR, V.le F. S. D’Alcontres 37, 98158 Messina, Italy; trusso@ipcf.cnr.it

**Keywords:** noble metal nanoparticles, pulsed laser ablation, surface enhanced Raman spectroscopy, antiepileptic drugs

## Abstract

The synthesis by pulsed laser ablation and the characterization of both the surface nanostructure and the optical properties of noble metal nanoparticle-based substrates used in Surface Enhanced Raman Spectroscopy are discussed with reference to application in the detection of anti-epileptic drugs. Results on two representative drugs, namely Carbamazepine and Perampanel, are critically addressed.

## 1. Introduction

Research in the field of nanomaterials opens scenarios that are anything but obvious. A particular spectroscopic technique, which aims at identifying and quantifying molecular species of biomedical interest becomes feasible when materials with specifically designed optical properties are available. In principle, it is possible to recognize a chemical species by means of the inelastic scattering of a probing laser light that discloses the features of the vibrational spectrum of the target molecule to be probed. The intrinsic limit of Raman spectroscopy is the very low scattering cross-section (about 10^−30^ cm^2^ molecule^−1^). This may be overcome when a *surface plasmon resonance* (SPR) and the associated strong increase of the scattered intensity is triggered at a nanostructured metal surface (Surface Enhanced Raman Spectroscopy, SERS). The wavelength and Full Width at Half Maximum (FWHM) of the SP, as recorded in the UV-Vis spectrum of the film, are the optical characteristics of a corrugated metal surface of specific interest when exploring its behavior as an active SERS substrate. Near the metal surface, the electric field enhancement associated with the exciting light is most relevant with the noble metals silver and gold. In particular, large electromagnetic field enhancements [1,2] are observed at *hot spots*, corresponding to specific local surface morphologies that include sharp tips, edges and thin interparticle gaps. The surface nanostructures of artificially roughened Ag and Au thin films display many hot spots [3] making them excellent SERS substrates. Thus, the goal is to synthesize Ag and Au films with surface nanostructure engineered so as to maximize the SERS signal.

Pulsed laser ablation (PLA) makes use of two alternative methods to produce artificially corrugated nanostructured surfaces. Both methods are based on the vaporization of a solid target by high-energy laser pulses in an ambient fluid, whose role is to confine the vaporized species and to induce their mutual aggregation, obtaining clusters and nanoparticles (NPs) without chemical precursors. The fluid can be a gas or a liquid transparent to the laser radiation.

**Figure 1:**
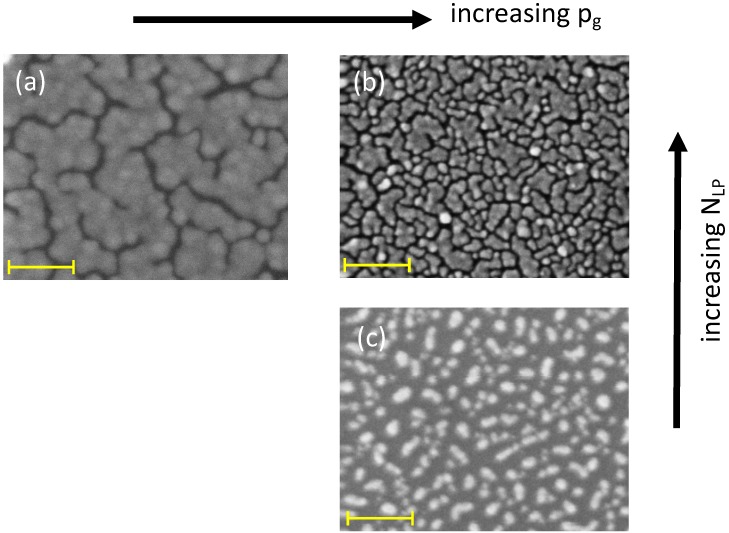
Scanning Electron Microscope (SEM) micrographs showing the dependence of the surface nanostructure of Au films deposited in Ar on the ambient gas pressure ($p_{g}$) and on the number of laser pulses ($N_{LP}$). (**a**) $N_{LP}$ = 3 × 10^4^, $p_{g}$ = 10 Pa; (**b**) $N_{LP}$ = 3 × 10^4^, $p_{g}$ = 100 Pa; (**c**) $N_{LP}$ = 5 × 10^3^, $p_{g}$ = 100 Pa. The yellow bars correspond to 100 nm. See Experimental for details.

When ablation is performed using an ns-laser (nanosecond-laser) in a high pressure, neutral ambient gas, NPs are directly deposited onto appropriate inert supports where they self-arrange leading to qualitatively different surface nanostructures [4,5,6]. For defined experimental conditions [4], a path connects two extreme morphologies: isolated, sphere-like NPs, more or less crowded together, and a continuous metal film. The two relevant parameters to design the surface nanostructure of the growing film are the number of laser pulses ($N_{LP}$) and the ambient gas pressure ($p_{g}$). In fact, for a progressively increasing $N_{LP}$ and $p_{g}$, we observe first particles more and more coalesced together whose size increases progressively while the shape departs from spherical and becomes more and more irregular. Later, islands separated by a network of randomly oriented channels with variable length and width in the few nm range develop. By increasing $p_{g}$, keeping constant $N_{LP}$ at low values, the support is covered by increasingly smaller, spherical NPs; at high $N_{LP}$ values, we observe islands with smaller average size and correspondingly a larger number of shorter channels (see Figure 1a,b). The $N_{LP}$ value defines the degree of support coverage. With increasing $N_{LP}$, keeping constant $p_{g}$, we observe a spatial densification of the NPs on the support. The role of $N_{LP}$ and $p_{g}$ is illustrated in Figure 1 for self-assembled Au NPs on (100) Si supports. The same qualitative trend is observed for Ag NPs.

Remarkably, for both metals, the average NP sizes observed in the Transmission Electron Microscope (TEM) agree with the predictions of a model for the plasma expansion through a high-pressure ambient gas [4,7]. Concerning the optical properties of the obtained nanostructures, for both metals, the SP wavelength decreases with increasing $p_{g}$ at fixed $N_{LP}$, as well as, when $p_{g}$ is kept fixed and $N_{LP}$ is increased, the SP wavelength increases.

When a liquid is used as the confining medium through which the ablation plasma expands, a colloidal solution is obtained. By pulsed laser ablation in liquid (PLAL), using a nano/picosecond laser source, surfactant-free NPs are produced in a single-step approach within a time scale of a few minutes [8]. In a stationary liquid, the process consists of the production of NPs by ablation of the target, and the contemporaneous fragmentation-assembling of dispersed NPs by continuous irradiation of already synthesized particles. The laser pulse duration is a relevant parameter for NP generation [9]. With picosecond pulses (ps pulses), the relevance of melting and thermal evaporation is strongly reduced with respect to nanosecond pulses (ns pulses). With short pulses, the ablation process becomes increasingly efficient involving a nearly instantaneous vaporization with minimized heat-affected zone [10], so that the colloidal solution is produced in a shorter time. Besides this, compared to ns pulses, ps pulses allow for mitigating primary plasma shielding that is detrimental to ablation efficiency [11].

Recently, in the scientific literature that deals with drug dosage [12], there emerged a promising technique based on Raman/SERS, specifically suited to low drug concentrations [13,14,15,16,17] that is complementary to those in clinical use. In a potential application scenario, it would be possible to trace the concentration of a drug in blood plasma samples of clinical origin subjected to limited and rapid treatments (e.g., centrifugation, extraction with solvents). The SERS measurement exploits the interaction of the drug molecule with a nanostructured metal surface and the intensity of the signal, under controlled conditions, allows for tracing the quantity of molecules adsorbed on the metal, which is a function of the drug concentration in the tested solution. The analytical capability of SERS was shown to be comparable with High-Performance Liquid Chromatography (HPLC) technique [18], thus making possible to study the potentiality of this spectroscopic technique in the field of Therapeutic Drug Monitoring (TDM). TDM is a clinical practice that involves determining the concentration of a drug in a biological fluid, usually blood plasma. This procedure is particularly relevant for drugs characterized by a narrow therapeutic index (NTI). In such drugs, the difference between the concentration at which therapeutic effects and the (slightly larger) concentration at which side effects are observed for the patient is minimal. In therapies that use NTI drugs, it is important to know the plasma concentration of the drug. This way, the patient’s clinical conditions can be associated with the required drug dose to guarantee the effectiveness of the treatment, avoiding the occurrence of side effects. TDM has been used for a long time in the clinical practice for NTI drugs. Among these drugs, we find anticancer drugs and antiepileptic drugs (AEDs). For the latter, toxicity can be induced by a small drug excess, whereas even a small reduction of the effective dosage can reduce the efficiency in controlling seizures.

Current research efforts in the SERS-TDM field point at overcoming the difficulties associated with the weakness of the signals of some drugs and/or to the background signal from much more abundant biomolecules coexisting with the drug in the fluid to be analyzed. A second relevant research direction is the development of SERS sensors (i.e., nanostructured metal surfaces) that combine a high sensitivity with spatial uniformity, control and reproducibility of the manufacturing process, not disregarding a low-cost production. Before the SERS technique can be routinely introduced into the clinical laboratory, besides the above technological issues, the treatment of the samples should also be optimized. Finally, validation procedures are required to develop quantitative SERS measurements. These imply determining drug concentration with standard reference methods (HPLC-MS or immunological assay).

In this work, we show how noble metal thin films resulting from self-assembled NPs can be produced in a controlled and reproducible way by PLA techniques. The obtained surface nanostructures are irregular and non-periodic. The peculiar morphological features of such nanostructures allow the good stability and reproducibility of the SPR of the films and lead to optimal electromagnetic enhancements in SERS [2]. Such control on plasmonic properties is required for effective application of SERS in analytics [14,19]. We have employed these films in TDM, focusing on two AEDs (Carbamazepine—CBZ, Perampanel—PER). In the case of CBZ, we prove that a SERS substrate can be re-utilized at least five times by washing it with methanol. We also show the good linear dependence of the SERS signal vs. CBZ concentration in the range 2.5 × 10^−5^ M to 2.1 × 10^−4^ M. In the case of PER, we exploit the protonation mechanism of the drug by HCl, as suggested by observed changes of the C=O stretching transition between Raman and SERS, and UV-Vis data taken on PER in acidic conditions. This paves the way to the control of the chemical enhancement pathway in SERS, which is triggered by effective chemical interaction of the analyte and the noble metal surface.

## 2. Experimental

### 2.1. Production of Au Substrates by PLA in High-Pressure Inert Gas

Au films were prepared at room temperature in a vacuum chamber, starting from a base pressure lower than 10^−4^ Pa using a KrF excimer laser (wavelength 248 nm, pulse width 25 ns, repetition rate 10 Hz, incidence angle 45°) focused onto an elemental target (Au, 99.99%) mounted on a rotating holder. The films were deposited onto pieces of glass, or (100) Si placed in front of the target at a distance of 35 mm. The target holder was rotated to avoid cratering of the target surface under repetitive ablation. Ablation was performed in Ar atmosphere at 100 Pa, with $N_{LP}$ fixed at 2 × 10^4^, and the laser fluence (*f*) kept constant at *f* = 2.0 J· cm^−2^. Sample surface nanostructuring was observed by scanning electron microscopy (SEM) using a Zeiss Supra 40 field ion microscope (Carl Zeiss NV, Via Varesina 162, 20156 Milano, Italy). UV–vis spectroscopy measurements of SPR were performed with a PerkinElmer UV-Vis/NIR Lambda 750 spectrophotometer (PerkinElmer Italia Spa, Viale dell’Innovazione 3, 20126 Milano, Italy) over the range 190–900 nm.

### 2.2. Production of Ag Colloids by PLAL using Water

We carried out PLAL of an elemental target (Ag, 99.9%) in deionized water using the second harmonic (532 nm) of a laser operating at 100 kHz repetition rate with pulse width of 6–8 ps. Ablation was performed at *f* = 1.5 J· cm^−2^, for an irradiation time of 10 min. The laser beam was focused with a galvanometric scanner to a spot of about 80 μm in diameter on the surface of the target that was scanned on a 10 × 10 mm^2^ area with a scan speed of 800 mm· s^−1^. The colloids were transferred on glass, or (100) Si supports by an ultrasonic spray-casting deposition method. The experimental setup consists of a deposition chamber equipped with an ultrasonic atomizer (Sonics VCX 130 W, Sonics & Materials Inc., 53 Church Hill Rd, Newtown, CT 06470, USA), a heated substrate holder and a system to remove excess vapors, thus guaranteeing standard and reproducible conditions. By ultrasonic spraying, we deposited a fraction of the produced colloids on nickel grids to perform Scanning Transmission Electron Microscopy (STEM), using an instrument operating at the primary accelerating voltage of 30 kV, at a working distance of 4 mm (Zeiss model Merlin Gemini 2).

**Figure 2:**
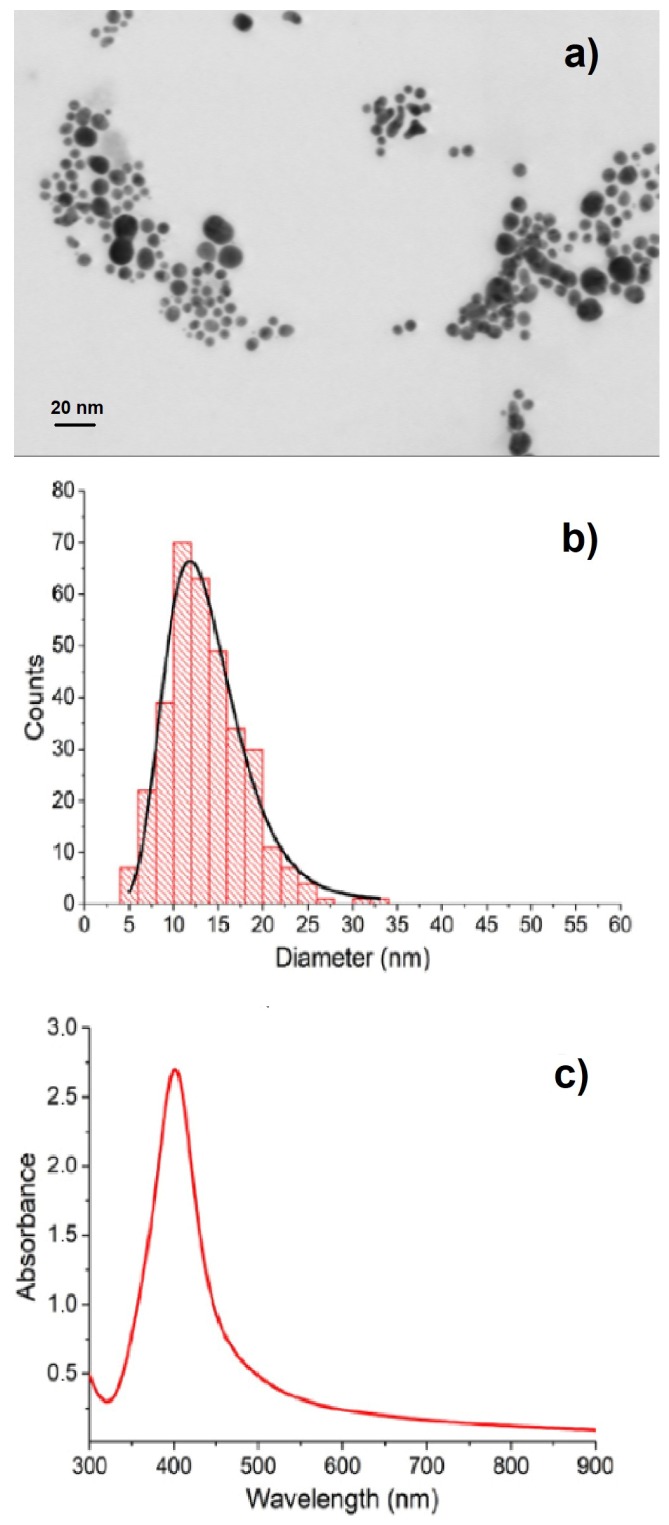
(**a**) Scanning Transmission Electron Microscope (STEM) images; (**b**) size distribution of nanoparticles (NPs); (**c**) optical absorbance spectrum of Ag colloids prepared by picosecond pulsed laser ablation in liquid (ps-PLAL) at the laser fluence of 1.5 J · cm^−2^.

By the same ultrasonic spraying procedure, we deposited Ag NPs onto (100) Si supports, obtaining substrates suitable for SERS measurements. In Figure 2, we show a representative STEM image (a), the average size distribution (b), and the optical absorbance spectrum (c) of Ag colloids prepared by ps-PLAL in water at the optimized laser fluence *f* = 1.5 J· cm^−2^. Nearly spherical NPs, whose size is about 15 nm, result from the likely agglomeration and overlap of smaller NPs (see Figure 2a). The UV-Vis absorption spectrum (Figure 2c) displays a narrow SPR, as expected on the basis of the narrow size distribution of the constituent NPs. This is the outcome of the optimization of the laser fluence. In the range 0.5 J· cm^−2^ ≤f≤ 1.5 J· cm^−2^, the SPR peak intensity increases and its FWHM decreases on increasing the laser fluence, keeping fixed all other deposition parameters.

### 2.3. Raman Spectroscopy

Raman and SERS spectra were collected by a HORIBA Jobin–Yvon LabRAM HR800 Raman Spectrometer(HORIBA France SAS, 231 rue de Lille, 59650 Villeneuve d’Ascq, France) with a solid-state laser (Laser XTRA, Toptica Photonics, TOPTICA Photonics AG, Lochhamer Schlag 19, 82166 Graefelfing (Munich), Germany) operating at 785 nm, equipped with a 600 grooves· mm^−1^ grating, a Peltier-cooled Charge-Coupled Device (CCD) detector, and notch filters to suppress Rayleigh scattering contributions. The same Raman spectrometer can be operated also with the 458 nm excitation from an Ar-ion laser.

## 3. Results and Discussion

The ability to produce nanostructured surfaces with a highly uniform morphology allows for designing sensors with a SERS signal of adequate reproducibility. This condition is mandatory when such sensors are used to detect analytes at low concentrations (as for drugs). In the recent past, we developed two complementary approaches to produce SERS sensors by exploiting the remarkable control in the production of nanoparticles offered by laser ablation techniques. The high control and reproducibility of these manufacturing processes allow the production of nanomaterials with optimized morphology, high sensitivity and spatial uniformity.

**Figure 3:**
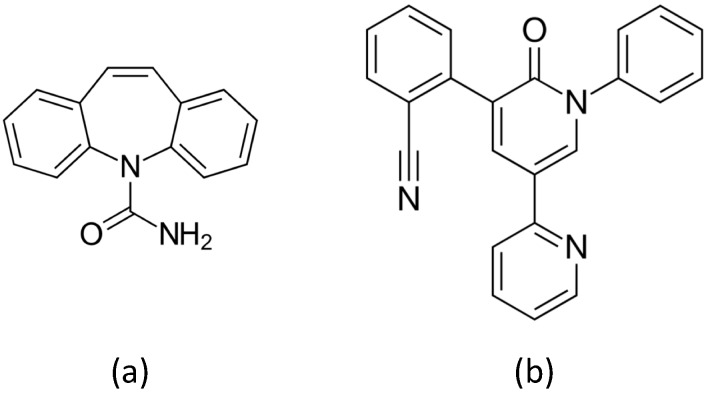
The chemical structure of (**a**) Carbamazepine, and (**b**) Perampanel.

The first approach discussed in Section 3.1 below employs ablation in high-pressure inert gas to produce Au sensors, which we tested on the AED Carbamazepine (Figure 3a). In the second approach, which we considered at a later time, Ag colloids are produced by ablation of the target in water, and are subsequently sprayed on a support to obtain the thin film SERS sensor. We show in Section 3.2 below our early and promising results obtained by testing these PLAL sensors on a second AED, namely Perampanel (Figure 3b). The choice of Ag allows for using HCl to control pH, fostering protonation of the drug, and providing chloride ions which are known to promote SERS action on silver [20]. Carbamazepine (CBZ) is a well-established drug largely used in developing Countries, whereas Perampanel (PER) is a new generation AED.

### 3.1. Quantitative SERS Detection of Carbamazepine

Nanostructured films made of arrays of NPs produced by Pulsed Laser Deposition (PLD) of a solid Au target in high-pressure inert gas and mutually assembled on an inert (glass) support behave as SERS sensors with good performances [16].

**Figure 4:**
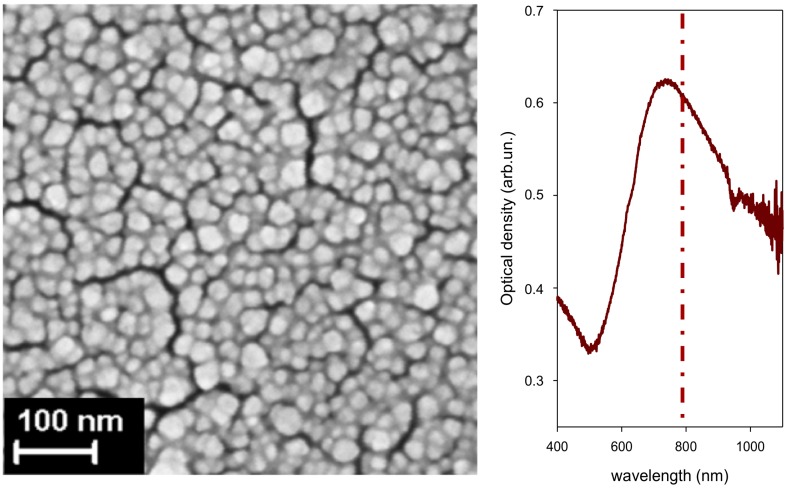
Surface nanostructure of an Au substrate deposited on (100) Si (ablation in Ar at 100 Pa, $N_{LP}$ = 2 × 10^4^). The pertinent UV-Vis spectrum with the position of the exciting laser radiation (785 nm) is reported.

In Figure 4, we show the surface nanostructure of an Au film deposited on (100) Si (ablation in Ar at 100 Pa; $N_{LP}$ = 2 × 10^4^). As shown in Figure 4, the SPR of films produced with this set of process parameters and deposited on glass matches the popular 785 nm laser excitation found in commercial Raman instruments, including portable ones. We used substrates of this kind, either deposited on glass, or on Si, throughout all investigations on CBZ. Part of the data we discuss on CBZ was presented in [6]. Here, we complete the data analysis including additional SERS markers, and we show in detail the evolution of SERS markers upon the washing procedure of a sensor.

**Figure 5:**
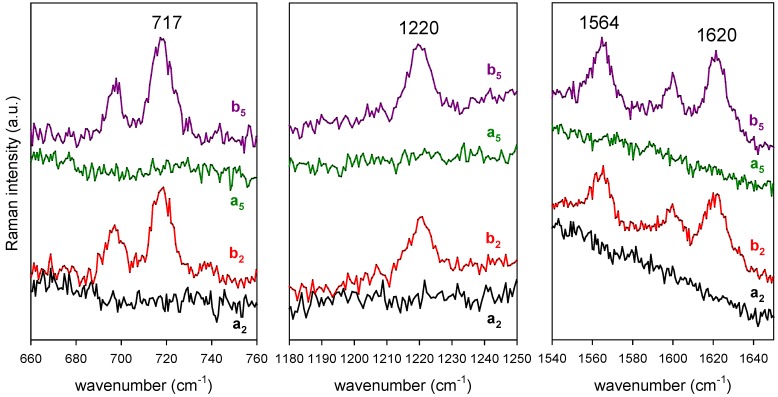
Effect of repeated immersions of a single Au SERS sensor (deposition on glass; ablation in Ar at 100 Pa, $N_{LP}$ = 2 × 10^4^) in a concentrated CBZ solution (100 mg/L in MeOH, 60 s immersion time) and subsequent washing with MeOH. The labels indicate the spectrum recorded after washing with MeOH (**a**) and after immersion in the CBZ solution (**b**). The pedix is the step number of this repeated sequence of experiments.

**Figure 6:**
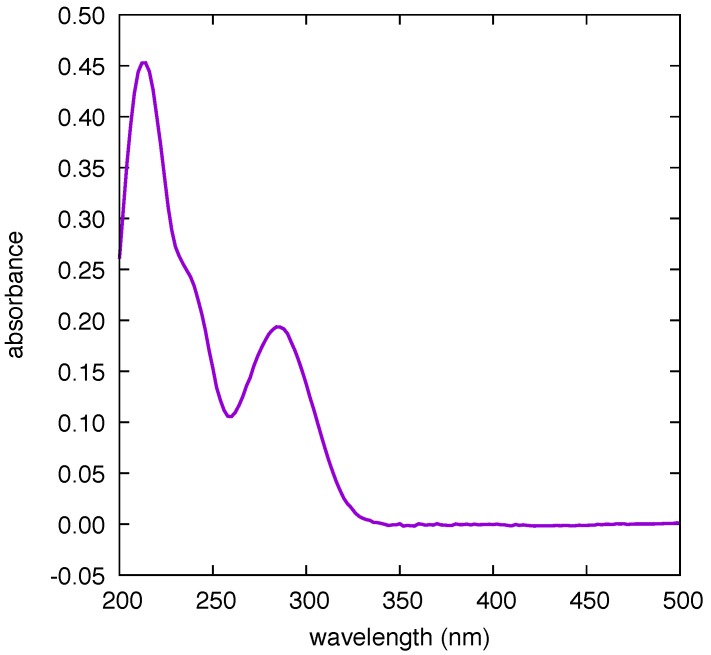
UV-Vis absorption spectra of a methanol solution of Carbamazepine (CBZ) (2.6 × 10^−5^ M, 1 cm pathlength quartz cuvette, Jasco V-570 spectrometer (Jasco Europe S.R.L., Via Luigi Cadorna 1, 23894 Cremella (LC), Italy)).

As reported elsewhere [16], there is agreement between the Raman and SERS features of CBZ over a wide wavenumber range. This is suggestive of a comparatively weak interaction between the Au substrate and the CBZ molecule. This fact, together with the remarkable stability of the substrates opens the way to recycle them, adopting a washing procedure with MeOH between consecutive measurements of drug concentration. The results of such washing procedure are shown in Figure 5. These results prove the following: (i) the background of the pristine sensor is blank in the spectral region of the CBZ markers; (ii) washing the sensor by immersion in MeOH for 5 min effectively removes the drug from the active surface as supported by the disappearance of the SERS markers; (iii) the successive immersion of the sensor in CBZ solution restores the initial SERS signal (i.e., the functioning of the sensor is preserved, even after five cycles of operation—which is enough to obtain the calibration curve of the sensor for four different values of concentration, see below); (iv) the interaction between CBZ and Au is weak (physisorption). Point (iv), together with the large wavelength distance between the absorption peak of CBZ (285 nm—see Figure 6) and the plasmon resonance (735 nm, very close to the laser excitation at 785 nm), indicate that the SERS of CBZ is electromagnetic in nature [21].

**Figure 7:**
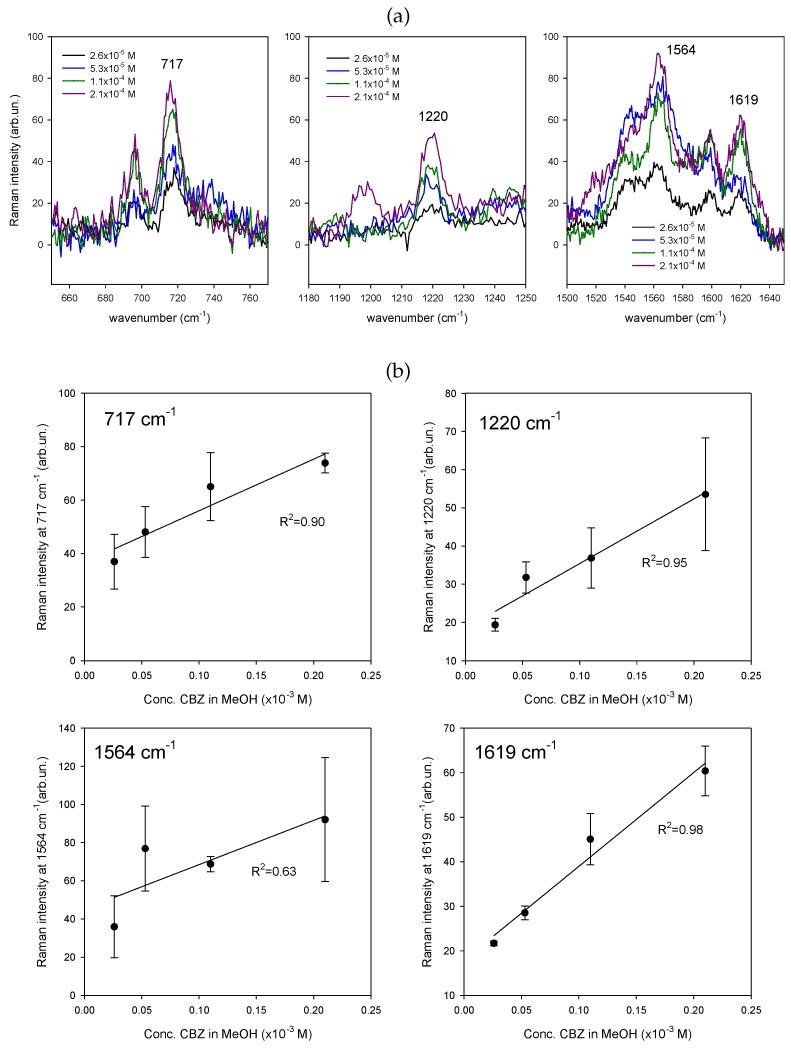
(**a**) average SERS spectra of CBZ in MeOH at different concentrations including the therapeutic range, in selected regions where the four CBZ markers are located (717, 1220, 1564 and 1619 cm^−1^); (**b**) calibration curves obtained by averaging the SERS intensities recorded at each peak vs. the corresponding CBZ concentration (range 2.5 × 10^−5^ to 2.1 × 10^−4^ M). Averages were done after baseline subtraction over a minimum of 3 to a maximum of 5 spectra, depending on concentration; vertical bars are the corresponding standard deviations.

After proving the re-usability of such SERS sensors, we quantified their response as a function of CBZ concentration in a range which includes the therapeutic range (2.5 × 10^−5^ M–5.1 × 10^−5^ M). Out of the seven available SERS markers [16], we selected those which show up more clearly from the background (717, 1220, 1564 and 1619 cm^−1^—see Table 1). The measurements shown in Figure 7 were performed by using *one single substrate* at increasing CBZ concentration. Spectra were recorded by taking 3 averages of 10 s each. The laser power at the sample surface was 1 mW over a spot area of 1 um in diameter. The substrate was dipped for 5 s in a 1 mL volume of the CBZ solution, and dried before recording. The substrate was washed in MeOH for 5 min after each SERS measurement. Control SERS spectra were taken after each washing procedure showing the complete disappearance of all CBZ features. The sensitivity to drug concentration is evident in Figure 7 for all the selected CBZ markers.

**Figure 8:**
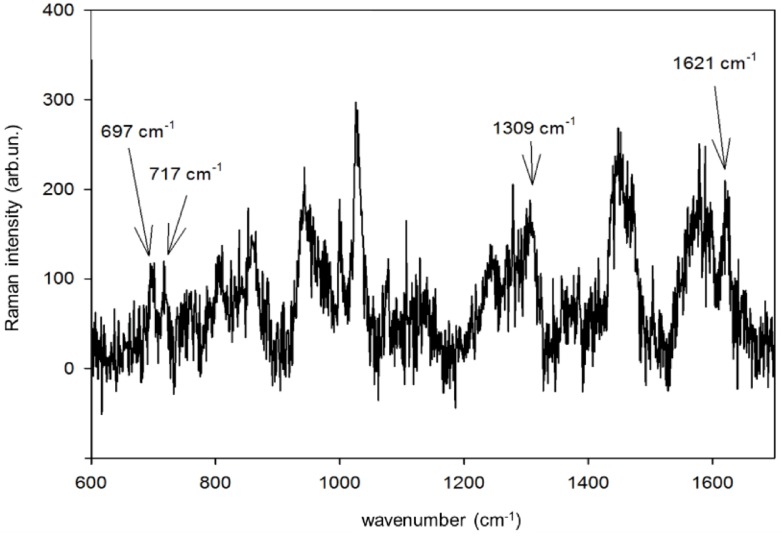
SERS spectrum of CBZ at the total concentration of 5.0 × 10^−5^ M in blood serum, recorded on an Au substrate deposited on (100)Si (Ar at 100 Pa, $N_{LP}$ = 2 × 10^4^).

In Figure 8, we report the SERS spectrum of CBZ at the total concentration of 5.0 × 10^−5^ M in blood serum from an epileptic patient. We used an Au substrate deposited on (100)Si to record the SERS spectrum adopting a higher laser power (10 mW) and a longer integration time (100 s). We observe four SERS signatures coincident with CBZ signatures, notwithstanding the large fraction (about 70%) of CBZ bound to albumin, as well as the complexity of a biological matrix such as the blood serum has. It is interesting to notice that the peculiar nanostructure of our SERS sensors produced by PLD in gas results in plasmonic hot spots localized at the narrow (few nm wide) channels that separate from each other Au islands made by agglomerated NPs on the support [2]. When samples are composed by molecules characterized by different molecular weights, and thus different diffusivities, this unique substrate morphology may enhance the probability that small molecules (such as drugs) reach the hot spots with respect to more bulky species.

### 3.2. SERS of Perampanel in Acidic Water Solutions

**Figure 9:**
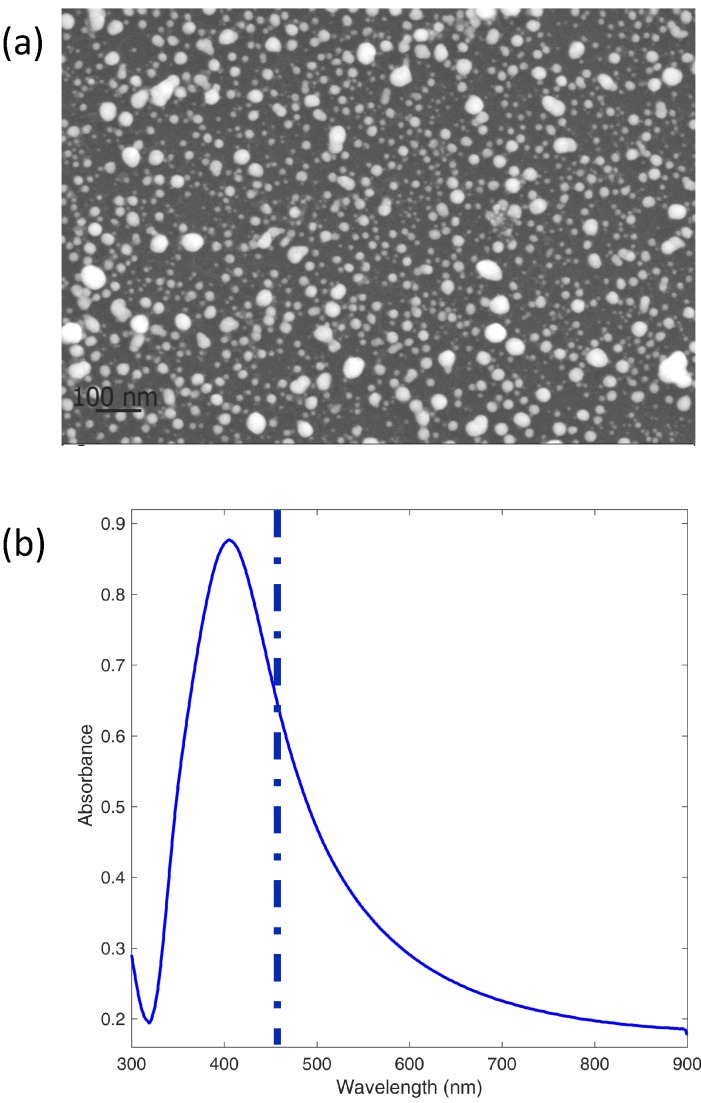
(**a**) surface nanostructure of an Ag substrate prepared by ps-PLAL at *f* = 1.5 J · cm^−2^ on a (100)Si support; (**b**) UV-vis spectrum taken on an analogous film sprayed on a glass support; the position of the exciting laser radiation is reported.

For SERS measurements of PER, we used an Ag substrate ultrasonically sprayed on a (100)Si support starting from colloids synthesized by ps-PLAL (see Experimental). The surface nanostructure (Figure 9a) consists of nearly spherical NPs in part coalesced to give spheroidal shapes and more complex agglomerates. The UV-Vis absorption spectrum from a companion film sprayed on glass is shown in Figure 9b.

Moving from our previous observation that an acidic environment (using HCl) fosters SERS on such Ag substrates through the protonation of PER, extracted from Fycompa^®^ tablets [22], we prepared aqueous solutions of PER (Cayman Chemical Item No. 23003; CAS 380917-97-5) at the concentration of 5 × 10^−5^ M at different pH values, starting from a concentrated methanol solution of PER in water acidified with HCl. The pH was checked each time before adding the drug. Our preparation procedure of PER solutions suggests that the charge state of PER plays a relevant role to SERS measurements, thus making evident the importance of the chemical enhancement mechanism [23].

**Figure 10:**
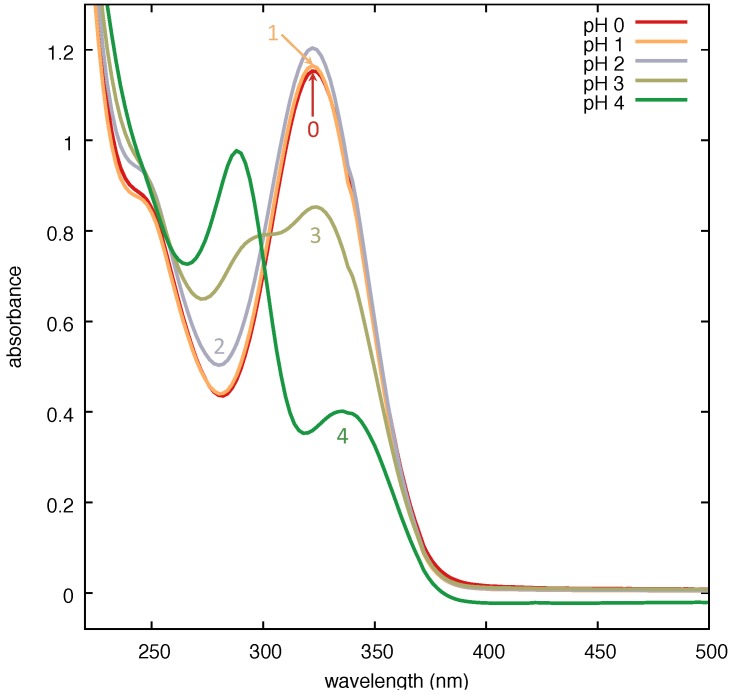
UV-Vis absorption spectra of aqueous solutions of Perampanel (PER) (5 × 10^−5^ M) prepared at various pH (1 cm pathlength quartz cuvette, Jasco V-570 spectrometer). The label on each curve corresponds to the pH value.

In Figure 10, we show that the absorbance of PER displays a systematic dependence on the pH of the solution. The protonation process appears to start at pH 3, and is achieved at pH 2 (and lower). We tested the SERS performance of the Ag substrate on the aqueous solution of protonated PER at pH 2. We prepared a 3 × 10^−4^ M aqueous solution of PER by mixing a suitable amount of a concentrated methanol solution of PER in water acidified with a mixture of HCl and H_2_SO_4_ in a 1:9 molar ratio. This expedient allows for limiting the quantity of Cl^−^ in the solution. Indeed, we verified that excessive amounts of Cl^−^ lead to the preferential formation of Ag-Cl surface bonds. The pH was checked before adding PER. The SERS spectrum was recorded with 458 nm excitation wavelength through a 50× microscope objective (NA = 0.75) at the solid–liquid interface formed by the solution droplet on the substrate, with 20 s exposure time (2 averages) and a laser power at the sample of 10 mW, on a spot area of 1 μm in diameter.

**Figure 11:**
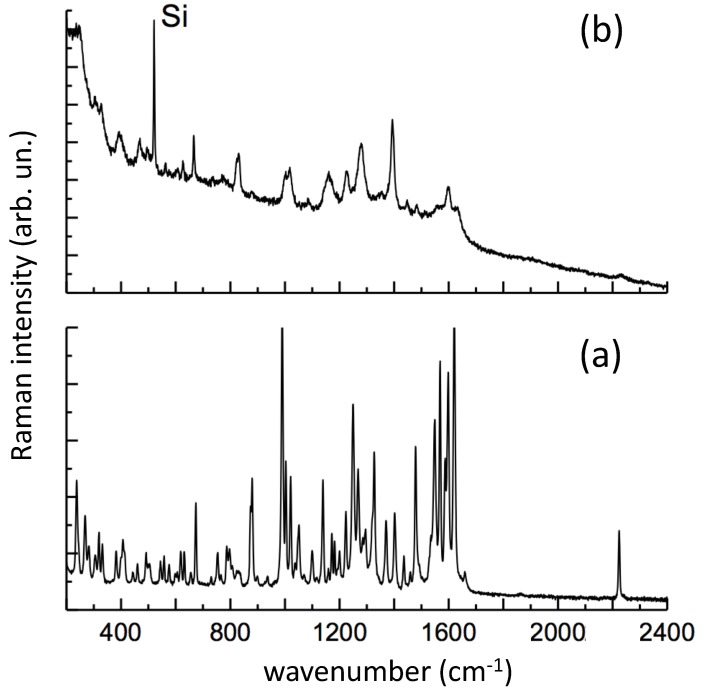
(**a**) Raman spectrum of solid Perampanel (PER) as received (785 nm excitation); (**b**) SERS spectrum of 3 × 10^−4^ M PER aqueous solution prepared at pH 2 with a mixture of HCl:H_2_SO_4_ in a 1:9 molar ratio. The SERS spectrum (458 nm excitation) was recorded at the solid–liquid interface of an Ag substrate prepared by ps-PLAL at *f* = 1.5 J · cm^−2^ on a (100)Si support.

In Figure 11, the SERS spectrum is compared with the Raman spectrum of solid PER, as received (laser excitation 785 nm). We observe that our early attempts to take SERS from *neutral* PER solutions failed. Remarkably, many of the major Raman features of solid PER appear in the SERS spectrum of an *acidic* PER solution (Figure 11). In particular, the following SERS lines of PER can be observed with a good signal to noise ratio: 666, 830, 877, 1000, 1018, 1158 (broad), 1225, 1279, 1394, 1447, 1483, 1514, 1599, 2231 cm^−1^. Most of these lines match with those recently measured for protonated PER on Au substrates produced by PLA [24], namely 670 (666, this work), 875 (877), 1000 (1000), 1135 (1158) cm^−1^.

Based on Density Functional Theory calculations (DFT B3LYP/6-31G(d,p)) [24], the principal Raman lines of PER can be assigned as in Table 2. The additional SERS lines of PER which can be detected with the Ag substrate produced by PLAL in this work are the following: 830, 1018, 1225, 1279, 1394, 1447, 1483, 1514, 1599, 2231 cm^−1^ (see Table 2 for details). Among these SERS signals, those assigned to a collective in-plane C-H bending, coupled with collective ring deformations, can be considered as a fingerprint of PER. These SERS signals find their correspondence in the Raman spectrum of the solid (1436, 1478, 1549, 1569, 1598, 1619 cm^−1^). The changes in relative intensity and the slight wavenumber shifts can be explained by the fact that SERS features belong to the protonated PER, whereas the Raman spectrum was recorded on the neutral species.

Notably, the weak C=O stretching peak of solid PER (1658 cm^−1^) disappears in the SERS spectrum of protonated PER. We deduce that the protonation of PER most likely occurs on the carbonyl.

## 4. Conclusions

We exploited the production by laser ablation of noble metal substrates engineered at the nanometer level with specific optical properties to perform SERS of selected AEDs toward a clinical application of the technique. An optimization of the substrate performance and the use of portable, possibly miniaturized Raman spectrometers are the next steps to address a point-of-care perspective.

## Figures and Tables

**Table 1:** Assignment of the SERS markers of Carbamazepine (CBZ) selected in this work (after [16]).

Wavenumber (cm^−1^)	Description
717	C-H out-of-plane bending at C=C and aryl groups
1220	C-H in-plane bending at C=C and aryl groups
1564	ring stretching
1619	C=C stretching

**Table 2:** Assignment of the markers of Perampanel (PER) discussed in this work (after [24]).

Wavenumber (cm^−1^)	Description
666	collective in-plane C-H bending
830	out-of-plane C-H bending
877	collective out-of-plane C-H bending of the three outer rings of PER
1000	trigonal ring deformation of the three outer rings of PER
1018	ring deformation
1158	collective C-H in-plane bending of the ring carrying the C≡N group
1225	in-plane C-H bending, ring deformation
1279	in-plane C-H bending, ring deformation of heterocycles
1394	in-plane C-H bending, central ring deformation
1447	in-plane C-H bending, pyridine ring deformation
1483	collective in-plane C-H bending coupled with collectve ring deformations
1514	ring deformation of the only phenyl group of PER, in-plane C-H bending
1599	ring stretching
2231	C≡N stretching

## Abbreviations

The following abbreviations are used in this manuscript:

AEDs	Antiepileptic Drugs
CBZ	Carbamazepine
FWHM	Full Width at Half Maximum
HPLC	High-Performance Liquid Chromatography
$N_{LP}$	number of laser pulses
NPs	nanoparticles
NTI	narrow therapeutic index
$p_{g}$	ambient gas pressure
PER	Perampanel
PLA	Pulsed Laser Ablation
PLAL	Pulsed Laser Ablation in Liquid
PLD	Pulsed Laser Deposition
SEM	Scanning Electron Microscopy
SERS	Surface Enhanced Raman Spectroscopy
SPR	Surface Plasmon Resonance
STEM	Scanning Transmission Electron Microscopy
TDM	Therapeutic Drug Monitoring
TEM	Transmission Electron Microscopy
